# l-Arginine-Dependent Epigenetic Regulation of Interleukin-10, but Not Transforming Growth Factor-β, Production by Neonatal Regulatory T Lymphocytes

**DOI:** 10.3389/fimmu.2017.00487

**Published:** 2017-04-25

**Authors:** Hong-Ren Yu, Ching-Chang Tsai, Ling-Sai Chang, Hsin-Chun Huang, Hsin-Hsin Cheng, Jiu-Yao Wang, Jiunn-Ming Sheen, Ho-Chang Kuo, Kai-Sheng Hsieh, Ying-Hsien Huang, Kuender D. Yang, Te-Yao Hsu

**Affiliations:** ^1^Department of Pediatrics, Chang Gung Memorial Hospital-Kaohsiung Medical Center, Graduate Institute of Clinical Medical Science, Chang Gung University College of Medicine, Taoyuan, Taiwan; ^2^Department of Obstetrics, Chang Gung Memorial Hospital-Kaohsiung Medical Center, Graduate Institute of Clinical Medical Science, Chang Gung University College of Medicine, Taoyuan, Taiwan; ^3^Department of Pediatrics, College of Medicine, National Cheng Kung University, Tainan, Taiwan; ^4^Allergy and Clinical Immunology Research (ACIR) Center, College of Medicine National Cheng Kung University, Tainan, Taiwan; ^5^Department of Pediatrics, Mackay Memorial Hospital, Taipei, Taiwan; ^6^Institute of Clinical Medicine, National Yang Ming University, Taipei, Taiwan; ^7^Institute of Biomedical Sciences, Mackay Medical College, New Taipei City, Taiwan

**Keywords:** l-arginine, neonate, regulatory T-cells, interleukin-10, DNA hypomethylation

## Abstract

A growing number of diseases in humans, including trauma, certain cancers, and infection, are known to be associated with l-arginine deficiency. In addition, l-arginine must be supplemented by diet during pregnancy to aid fetal development. In conditions of l-arginine depletion, T cell proliferation is impaired. We have previously shown that neonatal blood has lower l-arginine levels than adult blood, which is associated with poor neonatal lymphocyte proliferation, and that l-arginine enhances neonatal lymphocyte proliferation through an interleukin (IL)-2-independent pathway. In this study, we have further investigated how exogenous l-arginine enhances neonatal regulatory T-cells (Tregs) function in relation to IL-10 production under epigenetic regulation. Results showed that cord blood mononuclear cells (CBMCs) produced higher levels of IL-10 than adult peripheral blood mononuclear cells (PBMCs) by phytohemagglutinin stimulation but not by anti-CD3/anti-CD28 stimulation. Addition of exogenous l-arginine had no effect on transforming growth factor-β production by PBMCs or CBMCs, but enhanced IL-10 production by neonatal CD4^+^CD25^+^FoxP3^+^ Tregs. Further studies showed that IL-10 promoter DNA hypomethylation, rather than histone modification, corresponded to the l-arginine-induced increase in IL-10 production by neonatal CD4^+^ T cells. These results suggest that l-arginine modulates neonatal Tregs through the regulation of IL-10 promoter DNA methylation. l-arginine supplementation may correct the Treg function in newborns with l-arginine deficiency.

## Introduction

l-Arginine is a semi-essential amino acid; it can be synthesized from glutamine, glutamate, and proline by enzymes in the human intestinal–renal axis, but must be supplemented in the diet at times of physiological or pathological stress, including during pregnancy to aid fetal development (1). Both experimental and clinical studies have shown that certain human conditions, such as infertility, poor fetal growth, necrotizing enterocolitis in infants, cancer, trauma, and certain liver diseases, are associated with l-arginine deficiency (1–4).

l-Arginine can be metabolized to the cytotoxic and antimicrobial effector molecule nitric oxide through inducible nitric oxide synthase (iNOS). l-arginine can also be hydrolyzed by the enzyme arginase to ornithine and urea (1, 5), which depletes l-arginine. Arginase and iNOS compete for l-arginine. Induction of iNOS or arginase alone will result in reversible suppression of T cell proliferation. When both enzymes are induced, iNOS will generate peroxynitrites, which induce apoptosis of activated T cells. Therefore, relative changes in iNOS and arginase 1 activities may affect l-arginine metabolism and control specific types of T cell responses (6). l-arginine is also required for host defense against various pathogens and malignant cells. l-arginine modulates immune responses; it is critical for expression of the ζ-chain subunit of the T cell receptor complex, production of antibodies by B cells, and development of memory B cells (4, 7). Recently, l-arginine was also shown to regulate glycolysis and mitochondrial activity and enhance T cell survival and antitumor responses (8). Polymorphonuclear granulocytes and myeloid-derived suppressor cells have been shown to suppress T and natural killer cell proliferation and responses through arginase-mediated l-arginine depletion during activation (5, 9, 10). Moreover, dietary l-arginine supplementation in tumor-bearing or septic rats can increase thymus weight; interleukin (IL)-2/IL-2 receptor-mediated lymphocyte proliferation; cytotoxicity of T lymphocytes, macrophages, and natural killer cells; and delayed-type hypersensitivity responses (1).

Helper T cells are generally categorized based on their cytokine secretion profiles and their functions within the immune system (11–14). Th1 cells produce interferon-γ and play an important role in intracellular defense against microorganisms. Th2 cells produce IL-4, IL-5, and IL-13 and are responsible for allergic reactions and responses to parasitic infections (15). Regulatory T cells (Tregs), which produce IL-10 and transforming growth factor (TGF)-β, play key roles in the regulation of Th1/Th2-immune responses and peripheral tolerance (13, 14). Tregs suppress activated effector T cells and prevent pathological self-reactivity (16). They control effector T cell activation, proliferation, differentiation, and effector functions (17). Tregs can suppress effector T cells through the secretion of regulatory cytokines such as TGF-β and IL-10 (18). Other possible mechanisms of Treg-mediated T cell suppression include IL-2 deprivation *via* high surface CD25 expression and release of soluble CD25 (19). Although Tregs play a crucial role in immune responses, few studies have investigated their status and plasticity in neonatal blood.

Few reports have described l-arginine nutrition in neonates, especially preterm infants with l-arginine deficiency (20). We have previously shown that, compared to adults, neonates have lower plasma arginine levels and more abundant arginase 1 expression in leukocytes, which are associated with reduced lymphocyte proliferation (21). Additionally, we provided evidence that l-arginine modulates neonatal lymphocyte proliferation through an IL-2-independent pathway (22). Thus, l-arginine has distinct immune regulatory effects on neonatal and adult lymphocytes. In this study, we further investigated how l-arginine affects neonatal Tregs. Understanding the biological effects of l-arginine deficiency on T cell function may enable the design of novel treatments for neonatal immunodeficiency.

## Materials and Methods

### Collection of Human Umbilical Cord Blood (CB) and Adult Peripheral Blood

Human umbilical CB was collected at the time of elective cesarean section or normal spontaneous delivery from healthy mothers, after informed consent was obtained from the subjects as previously described (22). Adult peripheral blood samples were obtained from healthy volunteers aged 20–40 years. The leukocyte separation protocol has been described previously (23). In brief, leukocytes and red blood cells were separated from whole blood using 4.5% (w/v) dextran sedimentation. Polymorphonuclear cells and mononuclear cells (MNCs) were further separated by density gradient centrifugation with Ficoll-Paque™ (Amersham Pharmacia, Biotech AB, Uppsala, Sweden). This protocol was approved by the Institutional Review Board of Chang Gung Memorial Hospital, Kaohsiung Medical Center (104-7809C1) and the study was carried out in accordance with their recommendations.

### Induction of Cytokine Release by MNCs

We performed the cytokine induction protocol as previously described (24, 25). In brief, adult peripheral blood mononuclear cells (PBMCs) and cord blood mononuclear cells (CBMCs) (2 × 10^6^ cells/mL) were stimulated with or without purified phytohemagglutinin (PHA) (10 µg/mL), or 1 µg/mL anti-CD3 (HIT3a, Cat. #300314, BioLegend, San Diego, CA, USA) in combination with 1 µg/mL anti-CD28 (CD28.2, Cat. #302914, BioLegend), in 1-cm tissue culture plates with l-arginine-free medium (SILAC R1780 SIGMA, RPMI-1640 Medium) supplemented with 10% heat-inactivated fetal bovine serum, 1 mM glutamine, 1 mM sodium pyruvate, 50 mg/L l-leucine, 40 mg/L l-lysine, and 1× non-essential amino acids (Gibco cat. # 11140-035), 100 IE/mL penicillin, and indicated l-arginine (Sigma-Aldrich, St. Louis, MO, USA). After 72 h, cell-free culture supernatants were collected and assayed for cytokine production by enzyme-linked immunosorbent assay: TGF-β1 (R&D systems Inc., MN, USA) and IL-10 (R&D Systems).

### Isolation of CD4^+^ T Cells

CD4^+^ T cells were separated from MNCs using an IMag™ Cell Separation System (BD Biosciences, San Jose, CA, USA) as previously reported (26). In brief, MNC pellets were incubated with anti-human CD4 magnetic particles (BD Biosciences) for 30 min. Then, the labeled cells were resuspended in BD IMag™ buffer and isolated using a BD IMagnet™ (BD Biosciences).

### RNA Isolation and Real-time Reverse Transcription-Polymerase Chain Reaction (RT-qPCR) Analysis

Total RNA was extracted from cells using TRIzol^®^ Reagent (Invitrogen, Thermo Fisher Scientific, Waltham, MA, USA), according to the manufacturer’s instructions. Purified RNA was quantified by assessment of optical density at 260 nm using a NanoDrop^®^ ND-1000 spectrophotometer (Thermo Fisher Scientific) and qualitatively analyzed using a 2100 Bioanalyzer with RNA 6000 Nano Labchip Kit (Agilent Technologies, Palo Alto, CA, USA). RT-qPCR was then conducted as previously reported (22). In brief, a total of 200 ng of RNA was mixed with dNTPs and oligo-dTs (Invitrogen, San Diego, CA, USA) for 5 min at 65°C to reverse transcribe cDNA. The cDNA products were subjected to PCR amplification with specific primers (Table S1 in Supplementary Material), followed by SYBR Green quantification in an ABI Prism 7500 sequence detection system (Applied Biosystems, Foster City, CA, USA). For the relative quantification of gene expression, we employed the comparative threshold cycle (*C*_t_) method (27).

### Flow Cytometric Analysis of Intracellular FoxP3 and IL-10 Expression

We assessed the phenotype of Tregs by flow cytometry. CBMCs and PBMCs were cultured with PHA and the indicated concentration of l-arginine at 37°C for 48 h, then washed with fluorescence-activated cell sorting (FACS) buffer prior to evaluation of their Treg subsets. At the end of the stimulation, the cells were stained with anti-CD4-PerCP (BD Biosciences, Franklin Lakes, NJ, USA) and anti-CD25-FITC (Beckman Coulter, Brea, CA, USA) for 30 min, then fixed with paraformaldehyde/phosphate-buffered saline (PBS), and permeabilized using FACS permeabilizing solution (Sigma-Aldrich, St. Louis, MO, USA). For intracellular staining of FoxP3 and IL-10, cells were stained with anti-human FoxP3-PE (eBioscience, San Diego, CA, USA) and IL-10-APC (BD Biosciences). We analyzed the percentages of human CD4^+^CD25^−^, CD4^−^CD25^+^, and CD4^+^CD25^+^ cells, and the intracellular expression of FoxP3 and IL-10 in the subsets, using a FACSCalibur flow cytometer (BD Biosciences).

### Chromatin Immunoprecipitation (ChIP) Assay

Chromatin immunoprecipitation assays were performed as previously described (28). In brief, cell pellets (1 × 10^7^) were fixed with warmed 1% formaldehyde at 37°C, washed with ice-cold PBS, then assayed using an EZ-Magna ChIP™ A Kit (Millipore, Billerica, MA, USA), according to the manufacturer’s instructions. After the DNA was sheared to an average length of 200–1,000 bp by sonication, 5 µL of the supernatant (the input) was removed and stored at 4°C. The supernatant was then incubated overnight at 4°C with 5 µg of the indicated antibodies for immunoprecipitation. The antibodies used in the immunoprecipitation were as follows: acetyl histone H3 (Millipore), acetyl histone H3 lysine 4 (Cell Signaling, Danvers, MA, USA), monomethyl histone H3 lysine 4 (Abcam, Cambridge, UK), acetyl histone H3 lysine 9 (Cell Signaling), trimethyl-histone H3 lysine 36 (Abcam), and trimethyl-histone H3 lysine 4 (Abcam). The immunoprecipitated DNA was eluted and quantitated by RT-qPCR, performed at an annealing temperature of 57°C for 45 cycles with the indicated primers (Table S1 in Supplementary Material). The *C*_t_ values of the diluted input were adjusted to 100% of the input by subtracting 3.322 cycles (log 2 of 10) from the *C*_t_ value of the diluted input. One percent of starting chromatin was used as input. The amount of DNA precipitated by the indicated antibodies was calculated as percentage of the input using the following formula: % of input = 2Δ*C*_t_ × 100, where Δ*C*_t_ = *C*_t_ input—*C*_t_ IP.

### Genomic DNA Extraction, Bisulfite Conversion, and Pyrosequencing

Genomic DNA was isolated from CD4^+^ T cells using the PUREGENE^®^ DNA Purification Kit (Gentra Systems, Minneapolis, MN, USA), according to the manufacturer’s instructions. Bisulfite conversion of genomic DNA was performed using the EZ DNA Methylation™ Kit (Zymo Research, Orange, CA, USA), according to the manufacturer’s instructions. In brief, 500 ng of genomic DNA was added to M-Dilution Buffer, which was adjusted to a total volume of 50 µL with water, and incubated for 15 min at 37°C. CT Conversion Reagent was then added to the denatured DNA and incubated for 16 h at 50°C. We washed and eluted the DNA by centrifugation for 30 s, then performed PCR amplification of the target regions using a PyroMark PCR Kit (Qiagen, Valencia, CA, USA). Each PCR mix (25 µL) contained 2 × PyroMark PCR Master Mix, 10 × CoralLoad Concentrate, 5 µL Q-Solution, 5 µM primers, and 100 ng bisulfite-treated DNA. The cycling protocol was as follows: 95°C for 15 min, 45 cycles of 94°C for 30 s, 56°C for 30 s, and 72°C for 30 s, followed by a final extension of 72°C for 10 min. The biotin-labeled PCR products were captured by Streptavidin Sepharose high performance beads (Amersham Pharmacia). We purified the bead-bound PCR products, which were made single-stranded using a Pyrosequencing Vacuum Prep Tool (Qiagen). The sequencing primers were annealed to the single-stranded PCR products and pyrosequencing was performed using the PyroMark Q24 system (Qiagen). Quantitation of cytosine methylation was determined using PyroMark Q24 Software 2.0.6 (Qiagen). PCR amplification conditions were determined and sequencing primers were designed using PyroMark Assay Design Software (Qiagen). The primer sequences are listed in Table S1 in Supplementary Material.

### Statistics

Differences in parameters between PBMCs and CBMCs were analyzed using the Mann–Whitney *U*-test. Differences in parameters between different l-arginine conditions were analyzed using a one-way analysis of variance with Fisher’s least significant difference *post hoc* test. Data are expressed as the mean ± SEM. The correlation between IL-10 promoter DNA methylation levels and IL-10 mRNA levels in CB CD4^+^ T cells at the indicated concentrations of l-arginine was calculated as Pearson’s correlations. Differences with a *p*-value of less than 0.05 were considered statistically significant in all tests. All statistical tests were performed using SPSS 15.0 for Windows XP (SPSS, Chicago, IL, USA).

## Results

### Effects of l-Arginine on IL-10 and TGF-β Production by CBMCs

**Figure 1:**
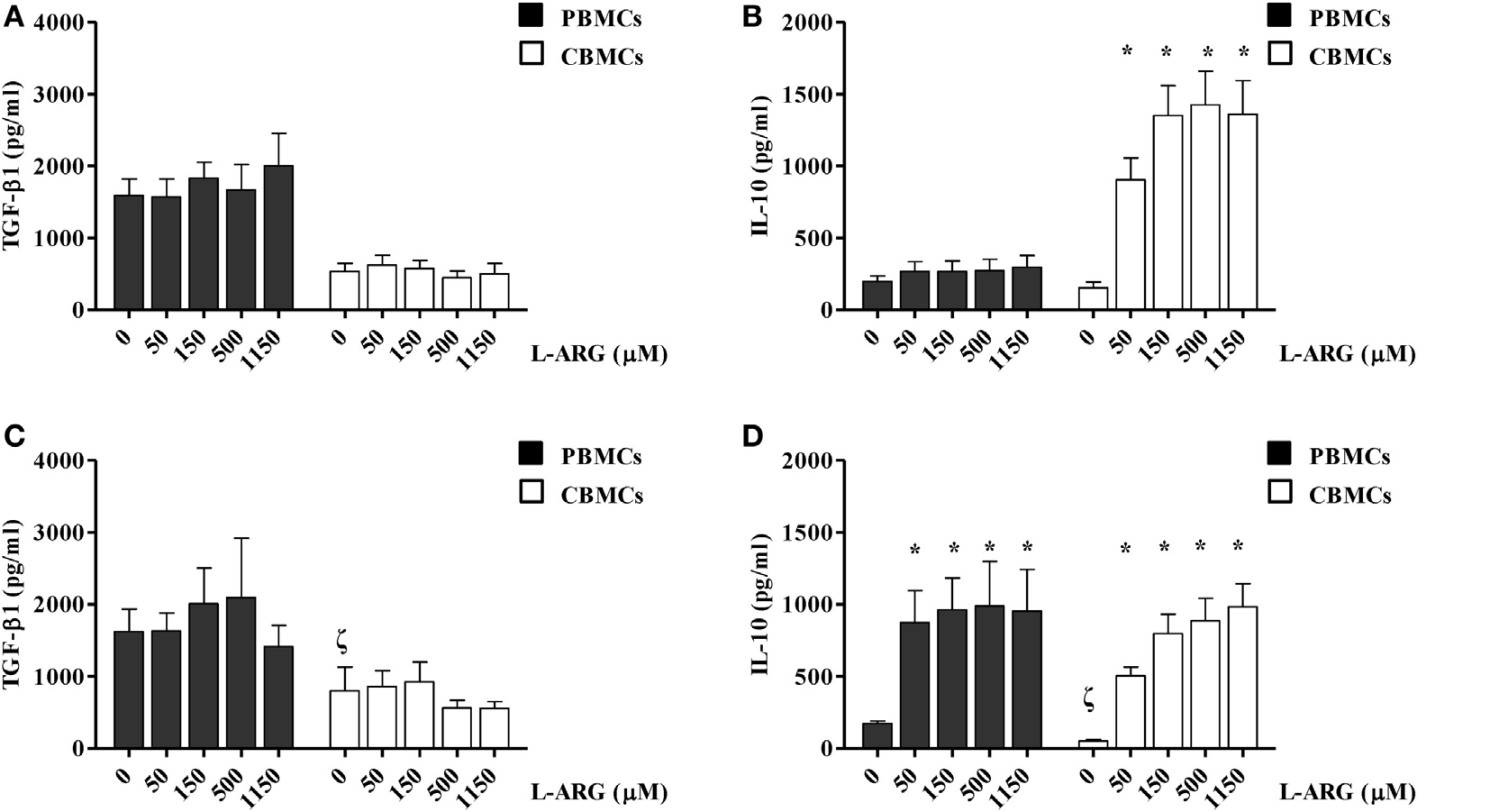
**Effects of l-arginine supplementation on transforming growth factor (TGF)-β and interleukin (IL)-10 production by adult and neonatal mononuclear cells (MNCs)**. MNCs isolated from adult peripheral blood and cord blood (CB) were suspended at a concentration of 2 × 10^6^/mL in 24-well plates and treated with 10 µg of phytohemagglutinin and the indicated concentrations of l-arginine for 72 h. The culture supernatants were collected and then levels of **(A)** TGF-β and **(B)** IL-10 were determined by enzyme-linked immunosorbent assay (ELISA). In a separate experiment, MNCs were treated with anti-CD3 (1 µg/mL) in combination with anti-CD28 (1 µg/mL) and the indicated concentrations of l-arginine for 72 h. The culture supernatants were collected and the levels of **(C)** TGF-β and **(D)** IL-10 were determined by ELISA (*n* = 6–9 for adult and 8–14 for CB as indicated). *p < 0.05 compared to the control with 0 µM l-arginine by ANOVA. ^ζ^*p* < 0.05 compared to the adult control without addition of l-arginine by Mann–Whitney *U*-test.

To clarify the modulatory effects of l-arginine on the Treg response, we determined the levels of TGF-β and IL-10 production by CBMCs and PBMCs, which were exposed to differential l-arginine levels in their respective origins, upon PHA stimulation. As shown in Figure 1, CBMCs produced less TGF-β1, but an equivalent amount of IL-10, compared to PBMCs in l-arginine-free culture medium. The addition of different concentrations of l-arginine had no effect on the production of TGF-β by CBMCs or PBMCs (Figure 1A). Thus, TGF-β production by human MNCs was l-arginine-independent (Figure 1A). Interestingly, exogenous l-arginine enhanced IL-10 production by neonatal, but not adult, MNCs in a dose-dependent manner (Figure 1B) upon PHA stimulation. Given that PHA stimulates leukocytes through CD2-mediated activation (29), we also used anti-CD3/anti-CD28 to specifically stimulate T cells (30). TGF-β1 production by PBMCs and CBMCs upon anti-CD3/CD28 stimulation was similar to that upon PHA stimulation. Anti-CD3/CD28-induced TGF-β1 production by human MNCs was also l-arginine-independent (Figure 1C). However, PBMCs and CBMCs behave in similar manner concerning the IL-10 production upon anti-CD3/CD28 stimulation (Figure 1D). The reason why CBMCs produce higher levels of IL-10 than PBMCs by PHA stimulation but not by anti-CD3/anti-CD28 stimulation may be because that PHA stimulation, which has been shown to mediate through CD2 activation (29), may be different from the combination of anti-CD3 and anti-CD28 stimulation (31). In order to explore the unique l-arginine-dependent PHA-induced IL-10 production of CBMCs, further studies were conducted on the cellular and molecular mechanisms of neonatal Tregs in CBMCs stimulated by PHA.

### l-Arginine-Induced IL-10 Production by Neonatal Tregs (Tregs)

**Figure 2:**
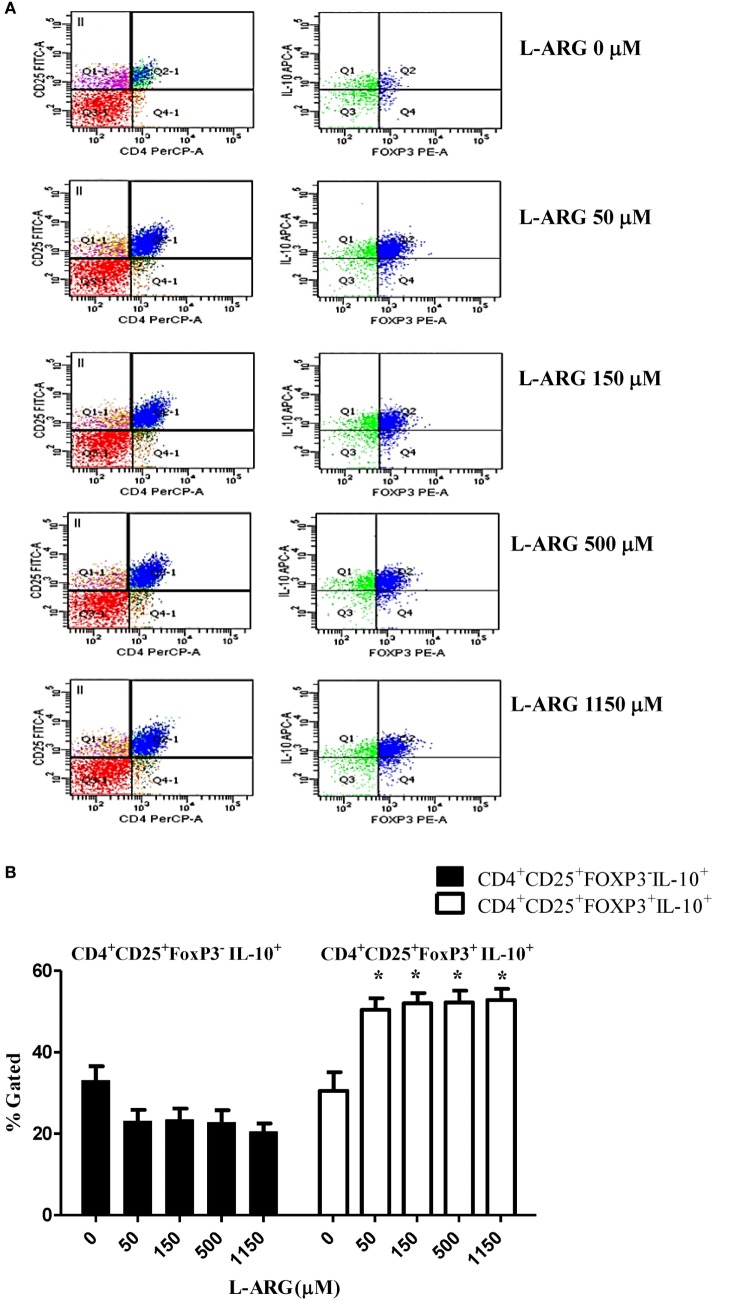
**Expression of intracellular FoxP3 and interleukin (IL)-10 in CD4^+^CD25^+^ T cells**. Mononuclear cells isolated from adult peripheral blood and cord blood were treated with 10 µg of phytohemagglutinin and the indicated concentrations of l-arginine for 48 h, then samples were stained with PerCP- and FITC-labeled antibodies specific for CD4 and CD25 cell surface markers, respectively. The cells were then permeabilized and stained with sheep antibodies specific for human FoxP3 and IL-10. **(A)** The flow plots show the effects of l-arginine supplementation on cell surface CD4 and CD25 expression in cord blood mononuclear cells, and the intracellular FoxP3 and IL-10 expression of CD4^+^CD25^+^ cells. The results are representative of four replicate experiments. **(B)** The bars illustrate the percentage of CD4^+^CD25^+^FoxP3^−^IL-10^+^ cells and CD4^+^CD25^+^FoxP3^+^IL-10^+^ cells in the presence of the indicated concentrations of l-arginine (*n* = 6 for each group). *p < 0.05 compared to the control without l-arginine by analysis of variance.

To study the modulatory effects of l-arginine on Tregs, we assessed the expression of cell surface CD4 and CD25, and intracellular expression of the transcription factor FoxP3, in cells cultured in l-arginine. As shown in Table 1, neonates had a higher proportion of CD4^+^CD25^+^ lymphocytes than adults, and l-arginine supplementation enhanced the proportion of CBMCs expressing CD4 and CD25. We evaluated the expression of intracellular FoxP3 by flow cytometry. Exposure to l-arginine was associated with higher MFI expression of FoxP3 in neonatal CD4^+^CD25^+^ T cells (Figure 2A). In order to identify the cell population associated with IL-10 production, CD4^+^CD25^+^ cells were gated for intracellular FoxP3 and IL-10. As shown in Figure 2B, CD4^+^CD25^+^FoxP3^+^ cells were responsible for the majority of IL-10 production in CD4^+^CD25^+^ CBMCs that were supplemented with l-arginine.

**Table 1:** **CD4 and CD25 cell surface expression upon l-arginine treatment**.

PBMCs	CD4^+^CD25^−^	CD4^−^CD25^+^	CD4^+^CD25^+^
l-ARG 0 µM	6.95 ± 0.32	36.83 ± 7.73	15.58 ± 1.49
l-ARG 50 µM	7.08 ± 3.07	33.15 ± 9.83	14.45 ± 2.20
l-ARG 150 µM	6.98 ± 3.26	34.65 ± 10.30	14.33 ± 2.37
l-ARG 500 µM	6.43 ± 3.63	39.20 ± 9.51	14.85 ± 2.48
l-ARG 1,150 µM	6.55 ± 3.43	35.95 ± 10.00	14.25 ± 2.29

**CBMCs**	**CD4^+^CD25^−^**	**CD4^−^CD25^+^**	**CD4^+^CD25^+^**

l-ARG 0 µM	1.40 ± 0.46	35.13 ± 10.80	23.87 ± 7.62
l-ARG 50 µM	1.00 ± 0.54	39.30 ± 12.15	41.20 ± 12.14*
l-ARG 150 µM	0.77 ± 0.38	38.40 ± 11.50	48.93 ± 14.32*
l-ARG 500 µM	0.83 ± 0.41	38.90 ± 11.59	48.70 ± 14.18*
l-ARG 1,150 µM	0.80 ± 0.42	38.37 ± 11.40	48.67 ± 14.17*

*Analysis of CD4 and CD25 expression on peripheral blood mononuclear cells (PBMCs) and cord blood mononuclear cells (CBMCs) by flow cytometry*.

*p < 0.005 compared to expression with 0 µM l-arginine level by analysis of variance.

### l-Arginine-Induced IL-10 Production by Neonatal CD4^+^ T Cells Correlated with IL-10 Promoter Hypomethylation but Not Histone Modification

**Figure 3:**
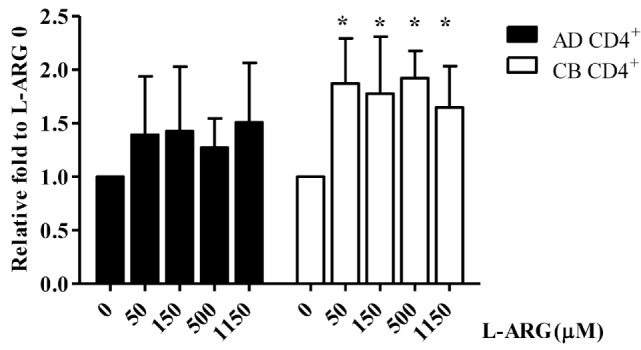
**Quantitative reverse transcription-polymerase chain reaction (RT-qPCR) analysis of interleukin (IL)-10 mRNA expression in CB and adult blood CD4^+^ T cells**. CD4^+^ T cells isolated from adult peripheral blood and CB were suspended at a concentration of 2 × 10^6^/mL in 24-well plates, then treated with 10 µg of phytohemagglutinin and the indicated concentrations of l-arginine for 48 h. Cell pellets were subjected to RT-qPCR analysis. IL-10 mRNA levels shown are relative to levels in 0 μM l-arginine (*n* = 6 for each group). AD CD4^+^, adult peripheral blood CD4^+^ T cells; CB CD4^+^, cord blood CD4^+^ T cells. *p < 0.05 compared to the control without l-arginine by ANOVA.

To investigate the mechanism of Treg IL-10 production, we treated isolated adult and CB CD4^+^ T cells with l-arginine and PHA as indicated, and studied their expression of IL-10 mRNA. As shown in Figure 3, consistent with IL-10 protein production, CB CD4^+^ T cells had significantly higher IL-10 mRNA levels in l-arginine-supplemented conditions than in the non-supplemented condition. However, l-arginine supplementation did not significantly enhance IL-10 mRNA expression by adult CD4^+^ T cells.

**Figure 4:**
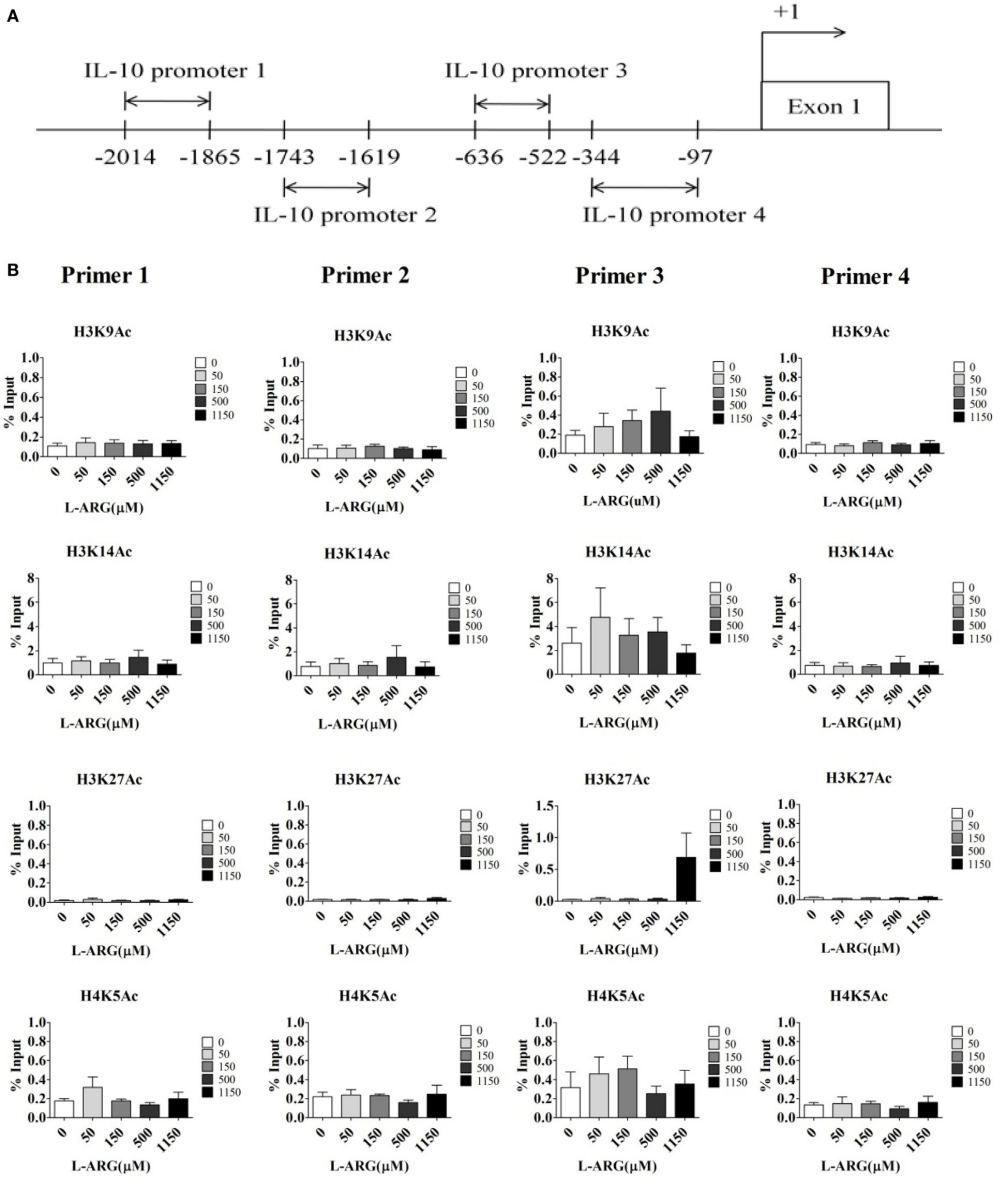

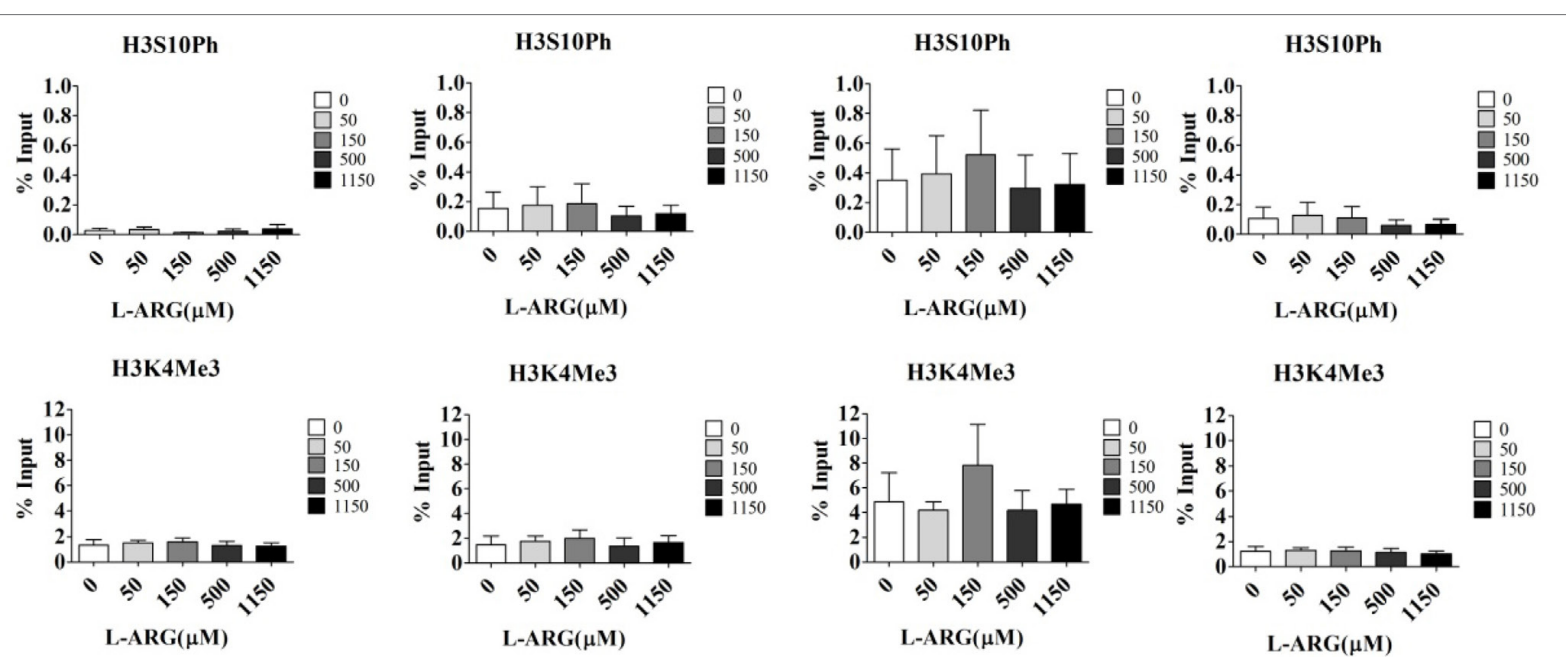
**Assessment of histone activation markers at the interleukin (IL)-10 promoters in cord blood (CB) CD4^+^ T cells with and without l-arginine supplementation**. CD4^+^ T cells isolated from CB were treated with 10 µg of phytohemagglutinin and the indicated concentrations of l-arginine for 48 h. **(A)** The diagram indicates the primer positions in the IL-10 promoters; “+1” indicates a transcription initiation site. IL-10 promoter 1 was 2,014–1,865 bp upstream of the transcription site. **(B)** Chromatin in the cell pellet was immunoprecipitated using anti-acetyl-histone H3 lysine 9 (ac-H3K9), anti-ac-H3K14, anti-ac-H3K27, anti-ac-H4K5, anti-phospho-histone H3 serine 10 (ph-H3S10), or anti-trimethyl-histone H3 lysine 4 antibodies. The bar graphs show the levels of the indicated histone markers at the various IL-10 promoters. Results are expressed as percentage of the input (mean ± SEM; *n* = 4).

Given that DNA methylation and histone modification can influence the expression of IL-10 mRNA, we next studied the epigenetic regulation of IL-10. First, we investigated the effect of l-arginine treatment on IL-10 promoter histone modification. We performed ChIP assays on nuclear extracts of CB CD4^+^ T cells using antibodies directed against acetyl-histone H3 lysine 9 (Ac-H3K9), Ac-H3K14, Ac-H3K27, Ac-H4K5, phospho-histone H3 serine 10, and trimethyl-histone H3K4, which are promoter activation markers (32, 33). We then used RT-PCR to determine the amount of IL-10 promoter input DNA that was bound to each protein. We designed four pairs of IL-10 primers for RT-PCR analysis (Figure 4A). As shown in Figure 4B, there were no differences in IL-10 promoter binding to these active histone markers in any of the l-arginine treatment conditions. We also investigated if repression of histone modification altered l-arginine-induced IL-10 transcription. ChIP assays were performed on the nuclear extracts of CB CD4^+^ T cells using antibodies directed against H3K9me3 and H3K27me3 (34). As with the other histone markers, there were no differences in H3K9me3 and H3K27me3 association with the IL-10 promoters among the l-arginine treatment conditions (Figure S1 in Supplementary Material). Thus, we did not detect either activating or repressing histone modifications of the IL-10 promoters in CB CD4^+^ T cells with exogenous l-arginine treatment.

**Figure 5:**
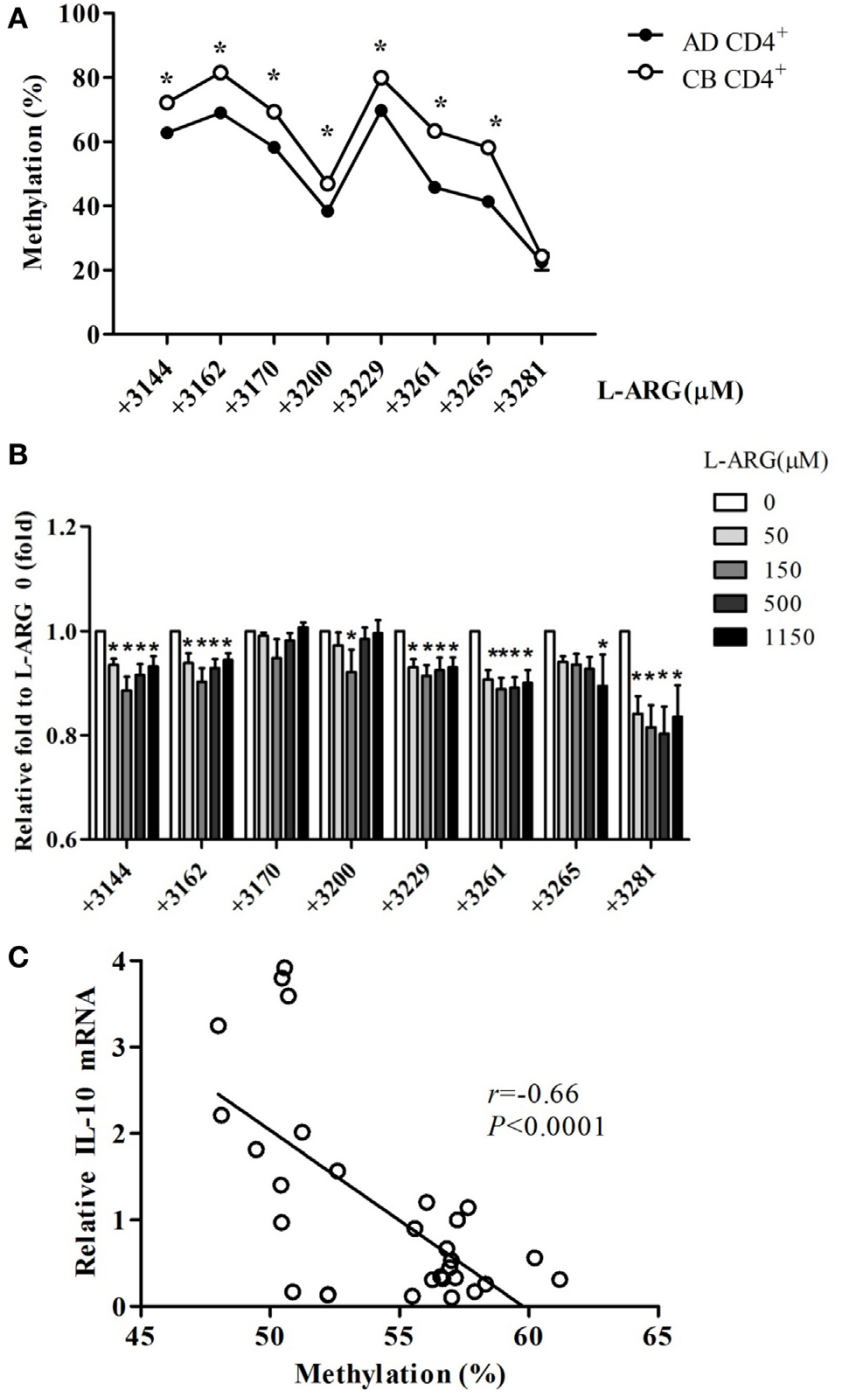
**Interleukin (IL)-10 enhancer methylation patterns in CD4^+^ T cells from adult and cord blood (CB)**. **(A)** The mean level of methylation for each of the eight CG pairs in intron 4 of the IL-10 locus in CD4^+^ T cells from adult (AD) and CB (*n* = 10). *p < 0.05 in a comparison between AD and CB by Mann–Whitney *U*-test. **(B)** The average level of methylation for the eight CG pairs (positions +3144, +3162, +3170, +3200, +3229, +3261, +3265, and +3281) in IL-10 intron 4 in CB CD4^+^ T cells with exposure to the indicated concentrations of l-arginine for 48 h (*n* = 6). *p < 0.05 compared to expression by cells treated with 0 μM l-arginine by analysis of variance (ANOVA). **(C)** The average levels of IL-10 promoter DNA methylation correlated with the relative IL-10 mRNA levels in CB CD4^+^ T cells at the indicated concentrations of l-arginine (*r* = −0.66; *p* < 0.0001). *p < 0.05 compared to the expression without addition of l-arginine by ANOVA.

To test whether changes in DNA methylation contributed to IL-10 expression in neonatal CD4^+^ T cells, we analyzed the methylation content of CG pairs in the 192-bp CpG island (position +3119 to +3310) within the intron 4 enhancer element of the IL-10 gene, which contains the transcriptional activator STAT5-binding site (35, 36). Figure 5A shows the average methylation content of eight CG sites in the IL-10 promoter from 10 CB and 10 adult CD4^+^ T cell samples. The position +3281 CG site was hypomethylated in both adult and CB CD4^+^ T cells. However, the methylation content of the other seven CG sites (positions +3144, +3162, +3170, +3200, +3229, +3261, and +3265) was significantly greater in CB CD4^+^ T cells than in adult CD4^+^ T cells. The methylation content of six of the CG sites (positions +3144, +3162, +3229, +3261, +3265, and +3281) decreased significantly in CB CD4^+^ T cells at the indicated exogenous l-arginine concentrations (Figure 5B). Moreover, we found that the average degree of methylation of the eight methylated CG pairs within the intron 4 CpG islands negatively correlated with the levels of IL-10 mRNA expression in CB CD4^+^ T cells (Figure 5C).

## Discussion

Although it is well known that l-arginine modulates T cell proliferation and immune responses, its mechanism of action is still not fully understood. Tregs can control improper activation, proliferation, and differentiation of effector T cells through several mechanisms in addition to the IL-2 signaling pathway (18). In previous studies, we have found that l-arginine modulates neonatal lymphocyte proliferation through an IL-2-independent pathway (22). In this study, we investigated how exogenous l-arginine enhances neonatal Tregs function in relation to IL-10 production and epigenetic regulation under PHA and anti-CD3/anti-CD28 stimulations. l-Arginine supplementation has been reported not to affect Treg frequency or IL-10 production by mouse splenocytes (37). To our knowledge, no experiments have focused on the modulatory mechanisms of l-arginine on human Tregs. In this study, we found that neonates, in contrast to adults, had impaired TGF-β, but not IL-10-related Treg responses. For both adults and neonates, the production of TGF-β1 was l-arginine-independent. IL-10 production by neonatal MNCs was mainly dependent on l-arginine-induced FoxP3 expression and hypomethylation of the IL-10 promoter in CB CD4^+^CD25^+^ T cells. Thus, we concluded that exogenous l-arginine modulates IL-10 production by neonatal Treg through demethylation of the IL-10 promoter.

The importance of l-arginine in the immune system was initially identified by the association of impaired T cell function and low-serum l-arginine levels in trauma patients (4). This low l-arginine-related T cell dysfunction could be rapidly reversed by l-arginine supplementation (4). l-Arginine depletion by arginase 1 and/or nitric oxide produced by granulocytes and myeloid-derived suppressor cells has been observed in certain cancers and infections (9, 38). The existence of myeloid-derived suppressor cells that produce arginase in cancer patients suggests that arginase production is a tumor evasive mechanism (39). l-Arginine depletion can result in inhibition of T lymphocyte proliferation, interferon-γ production, and CD3ζ downregulation, leading to impaired adaptive immune responses (7, 9). In addition to T lymphocytes, l-arginine depletion also suppresses the proliferation of natural killer cells and their production of IL-12/IL-18-mediated interferon-γ (5). Here, we provide evidence of l-arginine modulation of Tregs through epigenetic regulation. This is a novel finding, which may enable pharmacological regulation of Treg functions by inhibition of DNA methylation.

Interleukin-10 is an anti-inflammatory cytokine. It regulates innate and adaptive immune responses and limits immunopathologies (40). IL-10 is produced by many different immune cells, including dendritic cells, macrophages, and Tregs (40). Tregs include diverse immune cell populations that regulate the adaptive immune response. CD4^+^CD25^+^FoxP3^+^ T cells, natural Tregs (nTregs), are thymically derived. The two major types of induced Tregs are Treg type 1 cells and Th3 Treg cells (41). Treg type 1 cells have a CD4^+^CD25^+^FoxP3^−^ phenotype and specialize in IL-10 production (41, 42). Th3 cells express FoxP3 in the periphery and secrete TGF-β and IL-10 (41). Different conditions induce the various Treg subsets to produce IL-10. Induced Treg cells are known to be the major producers of IL-10 in filarial infections (43), whereas CD4^+^CD25^+^ Tregs produce IL-10 in *Leishmania major* and several other infections (40, 44, 45). In our study, we found that CD4^+^CD25^+^FoxP3^+^ cells were responsible for the majority of IL-10 production. There is no specific marker to differentiate nTregs from Th3 cells, as both show a CD4^+^CD25^+^FoxP3^+^ phenotype (41, 46); however, the IL-10-producing CD4^+^CD25^+^FoxP3^+^ T cells that responded to l-arginine supplementation in CBMCs were likely nTregs because Th3 differentiation is induced by TGF-β, and we did not observe a change in TGF-β production with l-arginine treatment.

In a previous report, we had illustrated that the l-arginine levels in CB plasma (about 50 μM) were less than those in adult plasma (about 100–150 μM) (22). CBMCs presenting overexpression of arginase significantly upregulated surface CD25, the IL-2 receptor, upon PHA stimulation after the exogenous supplementation with l-arginine (22). In this study, we have further determined that the increase of surface CD25 on CBMCs is mainly on CD4^+^ T cells, associated with higher IL-10 production through the regulation of IL-10 promoter DNA hypomethylation, but not histone modification. We also demonstrated that TGF-β1 production by PBMCs or CBMCs was l-arginine independent. The reason why CBMCs produce higher levels of IL-10 than PBMCs by PHA stimulation but not by the anti-CD3/anti-CD28 stimulation may be because that PHA stimulation, which has been shown to mediate through CD2 activation (29), may be different from the combination of anti-CD3 and anti-CD28 stimulation (31). Further studies to clarify the distinct signal transductions for IL-10 production by PBMCs and CBMCs between PHA and anti-CD3/CD28 stimulations are needed.

Our study is the first to show the effect of l-arginine on IL-10 promoter DNA methylation in neonatal CD4^+^CD25^+^ T cells, although the mechanism driving IL-10 DNA hypomethylation with l-arginine supplementation has not been determined. DNA methylation of CpG dinucleotides is an important epigenetic mechanism for gene regulation. Protein arginine methylation is a common posttranslational modification that regulates protein function in the cell. DNA methylation and protein arginine methylation are catalyzed by DNA methyltransferases and protein arginine methyltransferases, respectively (47, 48). Methyltransferases utilize S-adenosylmethionine (SAM) as a methyl group donor to form S-adenosylhomocysteine (SAH) and methylated substrates (including DNA and proteins). SAM treatment has been shown to increase global DNA methylation in human macrophages (49). Supplementation with l-arginine, a cofactor for SAM, can increase the production of SAH from SAM through the methylation-dependent generation of creatine (50). As SAH is a competitive inhibitor of SAM-dependent methyltransferases (51), l-arginine supplementation may cause IL-10 DNA hypomethylation through methyl group consumption and SAH production.

In conclusion, we studied the modulatory effects of l-arginine on Tregs in adults and neonates in depth. For both adult and neonatal MNCs, the production of TGF-β1 was l-arginine-independent. Neonatal MNCs produced higher levels of IL-10 than adult MNCs upon PHA stimulation. The frequency of IL-10-producing CD4^+^CD25^+^FoxP3^+^ T cells was increased by l-arginine supplementation in newborns. l-Arginine may modulate neonatal Treg IL-10 production through DNA hypomethylation. Given that Treg activation is a major mechanism for establishing immune regulation, l-arginine supplementation has the potential to correct the Treg function in newborns with l-arginine deficiency.

## Author Contributions

H-RY, T-YH, H-CH, KY, J-YW, H-CK, and L-SC contributed to designed the work; H-RY, C-CT, H-HC, J-MS, Y-HH, and K-SH contributed to data acquisition; H-RY, T-YH, L-SC, H-CH, H-CK, J-MS, and C-CT performed data analysis and interpretation; H-RY, KY, J-YW, and K-SH drafted the manuscript; H-RY, C-CT, Y-HH, and T-YH finalized the article. All authors have read and approved the final manuscript and agreed to be accountable for all aspects of the work.

## Conflict of Interest Statement

The authors declare that the research was conducted in the absence of any commercial or financial relationships that could be construed as a potential conflict of interest.

## Supplementary Material

Figure S1 **Assessment of histone repression markers at the interleukin (IL)-10 promoter in cord blood (CB) CD4^+^ T cells with and without l-arginine supplementation**. CD4^+^ T cells isolated from CB were treated with 10 µg of phytohemagglutinin and the indicated concentrations of l-arginine for 48 h. The primer positions in the IL-10 promoters are shown in Figure 4. Chromatin from the cell pellet was immunoprecipitated using anti-trimethyl-histone H3 lysine 9 (H3K9me3) or H3K27me3 antibodies. The bar graphs show the levels of histone activation markers at the different IL-10 promoters. Results are expressed as percentage of the input (mean ± SEM; *n* = 4).

## References

[1] Wu G Bazer FW Davis TA Kim SW Li P Marc Rhoads J Arginine metabolism and nutrition in growth, health and disease. Amino Acids (2009) 37(1):153–68. 10.1007/s00726-008-0210-y 19030957 PMC2677116

[2] Zamora SA Amin HJ McMillan DD Kubes P Fick GH Butzner JD Plasma l-arginine concentrations in premature infants with necrotizing enterocolitis. J Pediatr (1997) 131(2):226–32. 10.1016/S0022-3476(97)70158-6 9290608

[3] Li P Yin YL Li D Kim SW Wu G. Amino acids and immune function. Br J Nutr (2007) 98(2):237–52. 10.1017/S000711450769936X 17403271

[4] Popovic PJ Zeh HJ III Ochoa JB. Arginine and immunity. J Nutr (2007) 137(6 Suppl 2):1681S–6S. 17513447 10.1093/jn/137.6.1681S

[5] Oberlies J Watzl C Giese T Luckner C Kropf P Muller I Regulation of NK cell function by human granulocyte arginase. J Immunol (2009) 182(9):5259–67. 10.4049/jimmunol.0803523 19380772

[6] Bronte V Serafini P Mazzoni A Segal DM Zanovello P. l-Arginine metabolism in myeloid cells controls T-lymphocyte functions. Trends Immunol (2003) 24(6):302–6. 10.1016/S1471-4906(03)00132-7 12810105

[7] Rodriguez PC Zea AH Culotta KS Zabaleta J Ochoa JB Ochoa AC. Regulation of T cell receptor CD3zeta chain expression by l-arginine. J Biol Chem (2002) 277(24):21123–9. 10.1074/jbc.M110675200 11950832

[8] Geiger R Rieckmann JC Wolf T Basso C Feng Y Fuhrer T l-Arginine modulates T cell metabolism and enhances survival and anti-tumor activity. Cell (2016) 167(3):829–42.e13. 10.1016/j.cell.2016.09.031 27745970 PMC5075284

[9] Munder M Schneider H Luckner C Giese T Langhans CD Fuentes JM Suppression of T-cell functions by human granulocyte arginase. Blood (2006) 108(5):1627–34. 10.1182/blood-2006-11-010389 16709924

[10] Munder M. Arginase: an emerging key player in the mammalian immune system. Br J Pharmacol (2009) 158(3):638–51. 10.1111/j.1476-5381.2009.00291.x 19764983 PMC2765586

[11] Del Prete G Human Th1 and Th2 lymphocytes: their role in the pathophysiology of atopy. Allergy (1992) 47(5):450–5. 10.1111/j.1398-9995.1992.tb00662.x 1485646

[12] Romagnani S T-cell subsets (Th1 versus Th2). Ann Allergy Asthma Immunol (2000) 85(1):9–18; quiz, 21. 10.1016/S1081-1206(10)62426-X 10923599

[13] Sakaguchi S Ono M Setoguchi R Yagi H Hori S Fehervari Z Foxp3+ CD25+ CD4+ natural regulatory T cells in dominant self-tolerance and autoimmune disease. Immunol Rev (2006) 212:8–27. 10.1111/j.0105-2896.2006.00427.x 16903903

[14] Raghupathy R. Pregnancy: success and failure within the Th1/Th2/Th3 paradigm. Semin Immunol (2001) 13(4):219–27. 10.1006/smim.2001.0316 11437629

[15] Wegmann M. Th2 cells as targets for therapeutic intervention in allergic bronchial asthma. Expert Rev Mol Diagn (2009) 9(1):85–100. 10.1586/14737159.9.1.85 19099351

[16] Bettelli E Carrier Y Gao W Korn T Strom TB Oukka M Reciprocal developmental pathways for the generation of pathogenic effector TH17 and regulatory T cells. Nature (2006) 441(7090):235–8. 10.1038/nature04753 16648838

[17] Sojka DK Huang YH Fowell DJ. Mechanisms of regulatory T-cell suppression – a diverse arsenal for a moving target. Immunology (2008) 124(1):13–22. 10.1111/j.1365-2567.2008.02813.x 18346152 PMC2434375

[18] Vignali DA Collison LW Workman CJ. How regulatory T cells work. Nat Rev Immunol (2008) 8(7):523–32. 10.1038/nri2343 18566595 PMC2665249

[19] Lindqvist CA Christiansson LH Simonsson B Enblad G Olsson-Stromberg U Loskog AS. T regulatory cells control T-cell proliferation partly by the release of soluble CD25 in patients with B-cell malignancies. Immunology (2010) 131(3):371–6. 10.1111/j.1365-2567.2010.03308.x 20518821 PMC2996557

[20] Neu J Arginine supplementation for neonatal necrotizing enterocolitis: are we ready? Br J Nutr (2007) 97(5):814–5. 10.1017/S0007114507691612 17408520

[21] Yu HR Kuo HC Huang HC Chen TY Huang LT Tain YL Identification of immunodeficient molecules in neonatal mononuclear cells by proteomic differential displays. Proteomics (2011) 11(17):3491–500. 10.1002/pmic.201100123 21751377

[22] Yu HR Kuo HC Huang LT Chen CC Tain YL Sheen JM l-Arginine modulates neonatal lymphocyte proliferation through an interleukin-2 independent pathway. Immunology (2014) 143(2):184–92. 10.1111/imm.12300 24697328 PMC4172135

[23] Yu X Hirono KI Ichida F Uese K Rui C Watanabe S Enhanced iNOS expression in leukocytes and circulating endothelial cells is associated with the progression of coronary artery lesions in acute Kawasaki disease. Pediatr Res (2004) 55(4):688–94. 10.1203/01.PDR.0000113464.93042.A4 14764920

[24] Yu HR Chang JC Chen RF Chuang H Hong KC Wang L Different antigens trigger different Th1/Th2 reactions in neonatal mononuclear cells (MNCs) relating to T-bet/GATA-3 expression. J Leukoc Biol (2003) 74(5):952–8. 10.1189/jlb.0902474 12960249

[25] Yu HR Chen RF Hong KC Bong CN Lee WI Kuo HC IL-12-independent Th1 polarization in human mononuclear cells infected with varicella-zoster virus. Eur J Immunol (2005) 35(12):3664–72. 10.1002/eji.200526258 16285008

[26] Yu H-R Hsu T-Y Huang H-C Kuo H-C Li S-C Yang KD Comparison of the functional microRNA expression in immune cell subsets of neonates and adults. Front Immunol (2016) 7:615. 10.3389/fimmu.2016.00615 28066425 PMC5165026

[27] Yu HR Tain YL Sheen JM Tiao MM Chen CC Kuo HC Prenatal dexamethasone and postnatal high-fat diet decrease interferon gamma production through an age-dependent histone modification in male Sprague-Dawley rats. Int J Mol Sci (2016) 17(10):1610 10.3390/ijms17101610 PMC5085643 27669212

[28] Yu HR Kuo HC Chen CC Sheen JM Tiao MM Chen YC Prenatal dexamethasone exposure in rats results in long-term epigenetic histone modifications and tumour necrosis factor-alpha production decrease. Immunology (2014) 143(4):651–60. 10.1111/imm.12346 24962734 PMC4253513

[29] O’Flynn K Russul-Saib M Ando I Wallace DL Beverley PC Boylston AW Different pathways of human T-cell activation revealed by PHA-P and PHA-M. Immunology (1986) 57(1):55–60. 2417941 PMC1453876

[30] Trickett A Kwan YL. T cell stimulation and expansion using anti-CD3/CD28 beads. J Immunol Methods (2003) 275(1–2):251–5. 10.1016/S0022-1759(03)00010-3 12667688

[31] Green JM Karpitskiy V Kimzey SL Shaw AS. Coordinate regulation of T cell activation by CD2 and CD28. J Immunol (2000) 164(7):3591–5. 10.4049/jimmunol.164.7.3591 10725714

[32] Zhang X Edwards JP Mosser DM. Dynamic and transient remodeling of the macrophage IL-10 promoter during transcription. J Immunol (2006) 177(2):1282–8. 10.4049/jimmunol.177.2.1282 16818788 PMC2643023

[33] Koch CM Andrews RM Flicek P Dillon SC Karaoz U Clelland GK The landscape of histone modifications across 1% of the human genome in five human cell lines. Genome Res (2007) 17(6):691–707. 10.1101/gr.5704207 17567990 PMC1891331

[34] Ho TH Park IY Zhao H Tong P Champion MD Yan H High-resolution profiling of histone h3 lysine 36 trimethylation in metastatic renal cell carcinoma. Oncogene (2016) 35(12):1565–74. 10.1038/onc.2015.221 26073078 PMC4679725

[35] Tsuji-Takayama K Suzuki M Yamamoto M Harashima A Okochi A Otani T The production of IL-10 by human regulatory T cells is enhanced by IL-2 through a STAT5-responsive intronic enhancer in the IL-10 locus. J Immunol (2008) 181(6):3897–905. 10.4049/jimmunol.181.6.3897 18768844

[36] Zhao M Tang J Gao F Wu X Liang Y Yin H Hypomethylation of IL10 and IL13 promoters in CD4+ T cells of patients with systemic lupus erythematosus. J Biomed Biotechnol (2010) 2010:931018. 10.1155/2010/931018 20589076 PMC2879555

[37] Cao Y Feng Y Zhang Y Zhu X Jin F. l-Arginine supplementation inhibits the growth of breast cancer by enhancing innate and adaptive immune responses mediated by suppression of MDSCs in vivo. BMC Cancer (2016) 16:343. 10.1186/s12885-016-2376-0 27246354 PMC4888479

[38] Singer K Gottfried E Kreutz M Mackensen A. Suppression of T-cell responses by tumor metabolites. Cancer Immunol Immunother (2011) 60(3):425–31. 10.1007/s00262-010-0967-1 21240484 PMC11029601

[39] Zea AH Rodriguez PC Atkins MB Hernandez C Signoretti S Zabaleta J Arginase-producing myeloid suppressor cells in renal cell carcinoma patients: a mechanism of tumor evasion. Cancer Res (2005) 65(8):3044–8. 10.1158/0008-5472.CAN-04-4505 15833831

[40] Couper KN Blount DG Riley EM. IL-10: the master regulator of immunity to infection. J Immunol (2008) 180(9):5771–7. 10.4049/jimmunol.180.9.5771 18424693

[41] Peterson RA. Regulatory T-cells: diverse phenotypes integral to immune homeostasis and suppression. Toxicol Pathol (2012) 40(2):186–204. 10.1177/0192623311430693 22222887

[42] Zeng H Zhang R Jin B Chen L. Type 1 regulatory T cells: a new mechanism of peripheral immune tolerance. Cell Mol Immunol (2015) 12(5):566–71. 10.1038/cmi.2015.44 26051475 PMC4579656

[43] Metenou S Dembele B Konate S Dolo H Coulibaly SY Coulibaly YI At homeostasis filarial infections have expanded adaptive T regulatory but not classical Th2 cells. J Immunol (2010) 184(9):5375–82. 10.4049/jimmunol.0904067 20357251 PMC3407820

[44] Belkaid Y Piccirillo CA Mendez S Shevach EM Sacks DL CD4+CD25+ regulatory T cells control *Leishmania major* persistence and immunity. Nature (2002) 420(6915):502–7. 10.1038/nature01152 12466842

[45] Uhlig HH Coombes J Mottet C Izcue A Thompson C Fanger A Characterization of Foxp3+CD4+CD25+ and IL-10-secreting CD4+CD25+ T cells during cure of colitis. J Immunol (2006) 177(9):5852–60. 10.4049/jimmunol.177.9.5852 17056509 PMC6108413

[46] Korn T Bettelli E Oukka M Kuchroo VK. IL-17 and Th17 cells. Annu Rev Immunol (2009) 27:485–517. 10.1146/annurev.immunol.021908.132710 19132915

[47] Laird PW. The power and the promise of DNA methylation markers. Nat Rev Cancer (2003) 3(4):253–66. 10.1038/nrc1045 12671664

[48] Grillo MA Colombatto S. S-adenosylmethionine and its products. Amino Acids (2008) 34(2):187–93. 10.1007/s00726-007-0500-9 17334902

[49] Pfalzer AC Choi SW Tammen SA Park LK Bottiglieri T Parnell LD S-adenosylmethionine mediates inhibition of inflammatory response and changes in DNA methylation in human macrophages. Physiol Genomics (2014) 46(17):617–23. 10.1152/physiolgenomics.00056.2014 25180283

[50] Loscalzo J. l-Arginine and atherothrombosis. J Nutr (2004) 134(10 Suppl): 2798S–800S; discussion 818S–9S. 15465788 10.1093/jn/134.10.2798S

[51] Esse R Rocha MS Barroso M Florindo C Teerlink T Kok RM Protein arginine methylation is more prone to inhibition by S-adenosylhomocysteine than DNA methylation in vascular endothelial cells. PLoS One (2013) 8(2):e55483. 10.1371/journal.pone.0055483 23408989 PMC3568140

