# Supplementary material for: l-Arginine-Dependent Epigenetic Regulation of Interleukin-10, but Not Transforming Growth Factor-β, Production by Neonatal Regulatory T Lymphocytes
Source: Front Immunol. 2017 Apr 25;8:487. doi: 10.3389/fimmu.2017.00487 (PMC5403834; doi:10.3389/fimmu.2017.00487)
Supplement: Supplementary file 1 [file Table_1.DOC]

Supplementary Table 1. The primer sequences used for quantitative polymerase chain reaction (qPCR) and Chromatin immunoprecipitation (ChIP) assay

**Primer sequences for RT-q**PCR

| IL-10 | sense | 5'- GGCGCTGTCATCGATTTCT -3' |
| --- | --- | --- |
|  | antisense | 5'- GCCACCCTGATGTCTCAGTT -3' |
| 18S | sense | 5'- GTAACCCGTTGAACCCCATT -3' |
|  | antisense | 5'- CCATCCAATCGGTAGTAGCG -3' |

**Primer sequences for** ChIP assay

| Promoter primer 1 | sense | 5’- GGAAGGAGAAAGGTGCAAGAAG -3’ |
| --- | --- | --- |
|  | antisense | 5’- CCTGCTAGAAACTAGTATGGAGCTAACTC -3’ |
| Promoter primer 2 | sense | 5’- GCGTGGGCCCTCTCATC -3’ |
|  | antisense | 5’- AACCTGCTGCCACTGGAGTT -3’ |
| Promoter primer 3 | sense | 5’- TACTGTAGGAAGCCAGTCTC -3’ |
|  | antisense | 5’- CTTAGGTCTCTGGGCCTTAG -3’ |
| Promoter primer 4 | sense | 5’- CCACAATCAAGGTTTCCCGGC -3’ |
|  | antisense | 5’- CCACAGCTGAGGGCCTCTGC -3’ |

**Primer sequences for** Pyrosequencing

| Amplification primer | sense | 5'- AATGAGTAGGAAGTTGGATTTTTATTTAA -3' |
| --- | --- | --- |
|  | antisense | Biotin-AAATTCACACCCCCTAATATTAACAC |
| Sequencing primer | sense | 5'- TTTATTTAATTTGGAGTTGGT -3' |
|  | antisense | 5'- AAAAGAAATGGGGTTTTATATTTT -3' |
