# Supplementary figures and images for: l-Arginine-Dependent Epigenetic Regulation of Interleukin-10, but Not Transforming Growth Factor-β, Production by Neonatal Regulatory T Lymphocytes
Source: Front Immunol. 2017 Apr 25;8:487. doi: 10.3389/fimmu.2017.00487 (PMC5403834; doi:10.3389/fimmu.2017.00487)

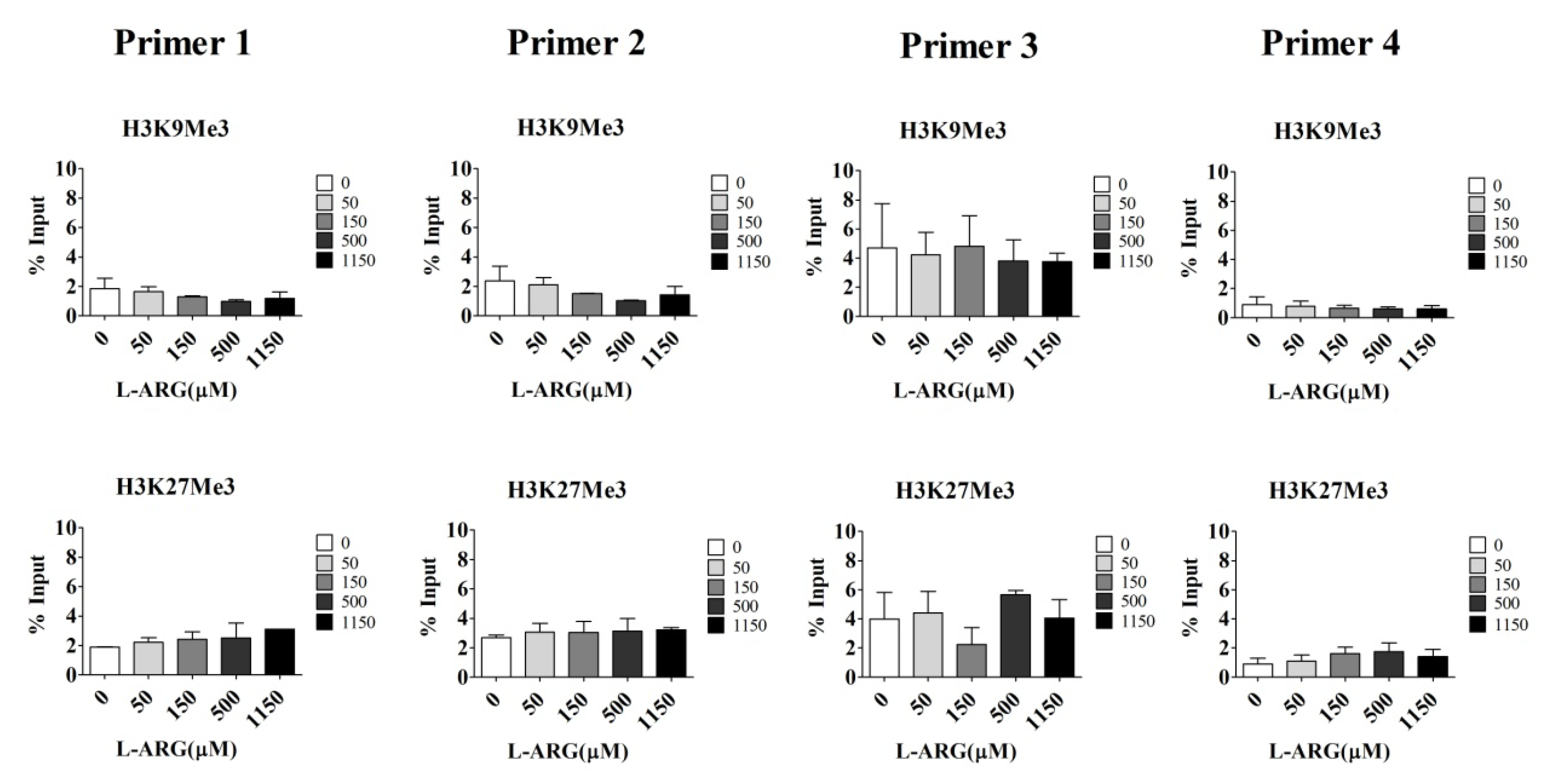

Supplement: Figure S1 — Assessment of histone repression markers at the interleukin (IL)-10 promoter in cord blood (CB) CD4+ T cells with and without l-arginine supplementation. CD4+ T cells isolated from CB were treated with 10 µg of phytohemagglutinin and the indicated concentrations of l-arginine for 48 h. The primer positions in the IL-10 promoters are shown in Figure 4. Chromatin from the cell pellet was immunoprecipitated using anti-trimethyl-histone H3 lysine 9 (H3K9me3) or H3K27me3 antibodies. The bar graphs show the levels of histone activation markers at the different IL-10 promoters. Results are expressed as percentage of the input (mean ± SEM; n = 4). [file Image_1.TIFF]
